# Convolutional Neural Network Approach for Multispectral Facial Presentation Attack Detection in Automated Border Control Systems

**DOI:** 10.3390/e22111296

**Published:** 2020-11-14

**Authors:** M. Araceli Sánchez-Sánchez, Cristina Conde, Beatriz Gómez-Ayllón, David Ortega-DelCampo, Aristeidis Tsitiridis, Daniel Palacios-Alonso, Enrique Cabello

**Affiliations:** 1 Departamento de Informática y Automática—Universidad de Salamanca, Avda. Fernando Ballesteros, 2, 37008 Salamanca, Spain; maraceli@usal.es; 2 Escuela Técnica Superior de Ingeniería Informática—Universidad Rey Juan Carlos, Tulipán, s/n, 28933 Móstoles, Madrid, Spain; cristina.conde@urjc.es (C.C.); beatriz.gomez@urjc.es (B.G.-A.); david.ortega.delcampo@urjc.es (D.O.-D.); aristeidis.tsitiridis@huawei.com (A.T.); enrique.cabello@urjc.es (E.C.)

**Keywords:** biometrics, presentation attack detection, Anti-spoofing, automatic border crossing systems, convolutional neural network, Bio-inspired systems

## Abstract

Automated border control systems are the first critical infrastructure point when crossing a border country. Crossing border lines for unauthorized passengers is a high security risk to any country. This paper presents a multispectral analysis of presentation attack detection for facial biometrics using the learned features from a convolutional neural network. Three sensors are considered to design and develop a new database that is composed of visible (VIS), near-infrared (NIR), and thermal images. Most studies are based on laboratory or ideal conditions-controlled environments. However, in a real scenario, a subject’s situation is completely modified due to diverse physiological conditions, such as stress, temperature changes, sweating, and increased blood pressure. For this reason, the added value of this study is that this database was acquired in situ. The attacks considered were printed, masked, and displayed images. In addition, five classifiers were used to detect the presentation attack. Note that thermal sensors provide better performance than other solutions. The results present better outputs when all sensors are used together, regardless of whether classifier or feature-level fusion is considered. Finally, classifiers such as KNN or SVM show high performance and low computational level.

## 1. Introduction

A current application of face verification systems in real environments with considerable security constraints is ABC systems [1,2]. These are used to verify passenger identities automatically through biometrics [3,4]. Due to this technology, border guards are helped by ABC systems to carry out routine tasks such as passport control and face verification at the international border-crossing points (BCP) of several countries in shorter times and with standard and homogeneous results. In addition, queue time delays are improved using these systems, which are mainly devoted to speeding up border crossings for “bona fide” travelers without perturbing the comfort of the passengers [5]. One of the most relevant tasks performed by ABC systems is traveler biometric identification. This function is often performed by comparing the captured subject face (denoted as an in situ picture) in a frontal static position with the face image stored in the chip passport (also known as eMRTD). In this configuration, the subject might remain in front of the camera.

In Europe, the Frontex agency has published technical guidelines and recommendations for ABC face verification procedures [6,7]. These guidelines are closely related to others international border agencies’ procedures. Standardization groups, both European and international, maintain highly harmonized levels and define the face verification procedure based on matching in the in situ image with the eMRTD image.

Currently, the performance of face verification systems has achieved remarkable results, but other aspects, such as robustness against attacks, are rarely considered or treated as relevant. An ABC is not an isolated system, and, usually, a border guard will supervise between six and twelve systems. In the case of ABC systems, security aspects and system vulnerabilities must be considered. Therefore, it is mandatory to eliminate or at least minimize them. In this sense, face presentation attacks are a popular topic whose relevance is increasing in the face biometrics area, and the number of references devoted to facial attack detection has grown in the last few years. This interest starts in research labs in which environmental conditions are different than real scenarios, and all found references are tested in these situations. The current approach is based on [8,9] projects in which close-to-real situations are achieved. Testing systems in a real border scenario is a challenging situation. Considering that passengers arrive in stressed conditions, they may change from different environmental temperatures (outdoor and indoor).

Attacks to the system, at the sensor level, are commonly labeled as presentation attacks [10,11], and preventing or detecting those attacks is denominated PAD [12]. PAD capabilities are extremely noteworthy in the design of ABC systems. Face verification is accomplished in an almost unsupervised scenario. BCP is a high-security environment, and border guards make efforts to guarantee that only passport holders cross the border. Identity impersonation is used in crossing lines by criminal organizations. Thus, automatic systems should be ready to process, not only bona fide travelers’ identities but also to detect presentation attacks to the ABC system.

Regarding Frontex’s recommendations, the maximum time which a passenger should spend on the border crossing process should not exceed 12 s [13]. At that point, the ABC system should perform three main specific tasks. Firstly, the passenger’s ID card is read and validated (usually a passport or official national document) with personal data stored in a chip. Secondly, it is verified if the traveler is the owner of the presented document. To carry out this action, it is mandatory to take and analyze the passenger’s face image in real-time. Then, this picture is compared with the stored image in the document chip. Thirdly, a query is submitted to the border control databases to check whether the traveler can or cannot enter the country. In a real ABC, the available moment to accomplish a presentation attack detection is while the traveler is waiting for the answer in the third task.

The methods presented in this study were carefully selected to fit at the available operational time and the standard computational power of an ABC system. Moreover, the sensors’ selection was a trade-off process because it is crucial to take into account the spectra of sensors, which should be as complementary as possible. For this reason, three kinds of sensors were considered: visible camera, near-infrared (NIR), and thermal. Each sensor reacts to different electromagnetic spectra: the visible wavelength was between 400 and 700 nm, the NIR wavelength was 800 nm, and the thermal wavelength was between 3 and 14 μm. Other factors should be considered, for instance, commercial sensors, which are easy-to-replace, and the acquisition time for the image by the sensor. In the present study, the underlying hypothesis is based on using several sensors to detect a plausible attacker. This sensors’ fusion provides a security high-level because the criminal has to circumvent, simultaneously, several sensors with different range spectra. Therefore, the attack is almost impossible to carry out.

According to the face biometric concepts, sensor level attacks are commonly produced with tools or artifacts to impersonate other people, using masks or printed pictures positioned in front of the camera sensor. In this sense, the sensor would capture the artifact instead of the person’s face. The ISO [12] defines as a presentation attack instrument (PAI) any biometric feature or object that is used in a presentation attack. It should be noted that the equipment necessary to attack systems is low-price and easy-to-acquire [14,15].

The relevance of these attacks can be explained at two levels: security and reputation costs. On the one hand, there are security risks related to criminals who are not detected crossing the border and accessing a country trouble-free. On the other hand, reputation costs are related to the possible diffusion of border-crossing videos in which criminals present different low-cost artifacts in social networks and the deep web, disseminating malicious information on the Internet.

In real scenarios, PAD attacks are based on processing only one image. In border crossing, a subject stands in front of the sentry or eGate without moving. The image is taken and processed while the system accesses official databases to check the subject’s identity. After that, the passenger can access or not access the country.

Currently, other kinds of sensors that work in a different spectrum range and can acquire multidimensional information are available on the market as well. The methodology presented in this study uses the distinct image sensors mentioned above (visible light camera, thermal and near-infrared sensors) for PAD. In an ABC system, visible light (VIS) images are presented to facilitate facial verification against the eMRTD image. Therefore, VIS images will be complemented with other acquisition systems, typically near-infrared (NIR) and thermal images. The main purpose of this research work is the use of isolated or mixed sensors, considering costs and computational constraints. The considered attacks are performed using the following artifacts: printed photo and mask and printed eyeless-mask, or an image displayed on a tablet.

Several methods to detect presentation attacks from images have been proposed for different research groups. Mainly, the approaches can be grouped into in situ, motion, texture-based, and context, based on visual inspections [10,11]. Note that the main characteristics selected are related to the texture and the color analysis following a classifier that can separate bona fide user images from attacks (dichotomic classification). The most remarkable trends are the use of deep learning and deep neural networks in processing and classification tasks in recent years. The solutions achieved in this study follow a novel approach for PAD systems, using a deep neural network, more specifically, convolutional neural networks (CNN) [16].

The paper is organized as follows. Section 2 is a review of related work. The method used is described in Section 4. A succinct explanation of the databased is described in Section 3, as well as a description of the algorithm and experiments. In Section 5, the results obtained are presented and discussed. In Section 6, the conclusions and future work are detailed.

## 2. Related Work

Artificial intelligence (AI) has become important in last years due to its vast real-world applications [17]. This section focuses on deep learning for facial PAD using multispectral images. A general state-of-the-art in the PAD area is out of the scope of this paper but a few examples of the current research include fingerprint PAD [18,19], makeup PAD [20], morphing and de-morphing PAD [21,22], etc. Note that the main problem of the presented approaches is that the databases considered were acquired in laboratory conditions [23]. However, real situations aggravate stress and physiological measures and emotions that change facial patterns (for instance, thermal images). Moreover, it is well known that attacker detection is easier when multiple systems are combined. Consequently, each acquisition system’s weakness is dampened by the other systems [24].

Kotwal et al. [25] explored the use of multispectral data (color imagery, near-infrared (NIR) imagery, and thermal imagery) for face PAD, specifically against the custom silicone mask attacks. Twenty-one custom made masks were used to establish baseline performance of several commonly used face-PAD methods, on the different imaging channels, using a new dataset (XCSMAD).

George et al. [26] proposed a multi-channel Convolutional Neural Network-based approach for PAD. They also introduced the new Wide Multi-Channel presentation Attack (WMCA) database for face PAD which contained a wide variety of 2D and 3D PA for both impersonation and obfuscation attacks. Data from different channels such as depth, thermal, color, and near-infrared were available to advance the study in face PAD. The proposed method was compared with feature-based approaches and found to outperform baselines, achieving an attack presentation classification error rate (APCER) of 0.3% on the introduced dataset.

In [27], the authors proposed a smart face liveness detector to prevent the biometric system from being “deceived” by the video or picture of a valid user that the counterfeiter took with a high definition handheld device (e.g., high-range tablet).

Albakri and Alghowinem [28] evaluated the liveness detection to suggest solutions that account for the weaknesses found in detecting spoofing attacks. They performed a proof-of-concept study to assess the liveness detection of 3D cameras in three devices, where the outcomes suggest that having more flexibility resulted in achieving a higher rate in detecting spoofing attacks.

In [29], the authors presented visible and NIR subsystems that were attacked in two ways. The first attack consisted of attacks with individual visible and NIR images (one by one). The second attack was composed of a multispectral system (VIS and NIR) that was attacked by pairs of visible and NIR images. In these kinds of attacks, the color of visible images is analyzed to detect a plausible attack, while the texture is checked to detect the attack in the case of NIR images. A multispectral system is based on a two-step verification process in which a color analysis is performed. The success rate presented was very high (100% in the same cases), but the type of attack tested was very simple, considering only static pictures.

Zhang et al. [30] proposed a method for PAD based on liveness evidence with specific acquisition devices. The authors used specific wavelengths to picture and store bona fide users and criminals. Therefore, specific devices were built and used to acquire spectral information. The reflectance obtained was used to train an SVM classifier. In addition, the authors achieved one-hundred percent accuracy when planar attack videos were used but 92% accuracy on planar attack photos versus bona fide users. When bona fide individuals were compared with mask face attacks, 89% accuracy was achieved. Then, the authors affirmed that their method depends on the material that covers the faces.

In [31], the authors’ approach consisted of distinguishing whether the analyzed object was a real person (denoted by liveness concept). A real person presents physiological features such as sweating, blood pressure, and temperature. However, in an artificial human, such as a mannequin, these kinds of characteristics are not presented. For this reason, the proposal was based on the gradient of the captured images by a multispectral system with different wavelengths. The approach was tested on a database including two-dimensional planar photos, 3-D mannequins, and masks. Regarding the classification task, the SVM classifier was used to learn the gradient-based features of genuine and fake faces. The authors reported a true positive rate (TPR) of 98.3% and a true negative rate (TNR) of 98.7%.

Currently, CNN is widely used in recognition and classification tasks, obtaining satisfactory results [32,33,34]. A canonical CNN structure for face antispoofing was implemented by Yang et al. [35]. The authors used videos recorded by a visible light camera. The CNN adopted was the well-known AlexNet (which won ImageNet 2012) [36]. The authors used the CASIA (formed by 55 users) and REPLAY-ATTACK (50 subjects) databases. The face was detected in each sample and cropped with different scale ratios. The authors asserted that, despite not carefully selecting the parameters of the CNN, the obtained results were successful (half total error rate (HTER) lower than 5%). However, the inter-test results did not have good performance.

A complex modification is a recurrent neural network (RNN) with long short-term memory (LSTM) units above a CNN architecture (based on AlexNet) [37]. The authors reported a 5.9% HTER and an equal error rate (EER) of 5.17% with the CASIA database (50 subjects: 20 for training and 30 for testing).

In [16], the authors implemented a CNN+RNN to detect presentation attacks based on depth maps from visible light images. The database, called Spoof in the Wild, was built by the authors and is composed of 165 subjects (including bona fide and attack videos). The attacks performed were printed images of different qualities and videos displayed on different devices. The CNN was used to obtain depth maps from the individual frames, and the RNN assessed the temporal features.

A new architecture was developed by Lucena et al. [38], which was denominated FASnet and based on the VGG-16 architecture [39]. The neural network was pretrained with the ImageNet database. The authors used the transfer learning approach, more specifically, fine-tuning, and used CNN to address face anti-spoofing methods. The authors obtained 100% accuracy and 0% HTER using the 3DMAD database. However, the performance decreased to 99.04% accuracy and 1.20% HTER using REPLAY-ATTACK.

Despite the numerical results shown in this section, it is difficult to compare in a straightforward way numerical outcomes accomplished in the literature. These drawbacks were manifested by Ramachandra and Busch [40].

## 3. Database Description

A new database named the “FRAV-Attack” database is developed in close-to-real scenarios to assess systems that should prevent presentation attacks. The main purpose of this database is to cover border-crossing presentation attacks with different facial representations provided by several sensors that can be placed in an eGate.

Regarding the data considered, the baseline face antispoofing databases are frequently addressed using visible light images. Therefore, the free-to-charge available face presentation attack databases (for research purposes) mainly contain RGB images. In recent years, more sensors have been incorporated into databases, but the acquisition process has always been performed under laboratory conditions such as the CASIA [41], REPLAY-ATTACK [42] and MFSD-MSU databases [14]. The acquisition process was carried out for several days, and users were volunteers who signed an informed consent form. In addition, the database follows the standards agreed with the RPGD regulation of the Spanish Agency belonging to the European Union. Moreover, the database is composed of 185 users. Sixty-two percent of users are male and 38% are female. Overall, 75% are between 18 and 40 years old and the remaining are older than 40 years. The samples were acquired under uniform and controlled illumination in a border scenario [8,9] in close proximity to the real situation.

The following sensors were used with each user: a visible light camera SONY ILCE-6000Y, a mobile phone camera with a FLIR ONE thermal sensor, and a surveillance camera HIKVISION. Each camera took several samples (bona fide users and criminals). Sensors have the following features. the thermal sensor has 160 × 120 pixel resolution, temperature measurements up to 120 ${}^{{}^{\circ}}$C, and can detect temperature differences down to 100 mK; the NIR sensor has an operating range (min–max) of 0.5–3.5 m and a depth resolution of 480 × 360 at 60 FPS; and the visual camera is a standard USB camera with common features.

Four kinds of artifacts are selected to attack the ABC systems. The attacks were selected taking into account which could be performed in a real operational situation for the border crossing at the airport queue. For this reason, artifacts which could be easily detected by border guards at a glance were discarded. All selected artifacts could be effortlessly put on and taken off because an attack should be carried out at a minimum time. Moreover, these artifacts could be hidden quickly in the luggage. Therefore, the use of 3D rigid masks could be a remarkable counterexample of that because these artifacts are bigger than a normal face and easily detectable, even if the mask’s color tries to imitate the human skin or the criminal wears a scarf, a cap, or sunglasses. Besides, a 3D mask is difficult to hide in luggage and its correct position is more intricate and requires more time than selected artifacts. Notice that flexible 3D masks improve some of the previous drawbacks but, again, proper mask collocation requires a lot of time. For the sake of brevity, only one real incident was detected, and the criminal was arrested for this kind of attack (flight from Hong Kong to Vancouver on 29 October 2010). It should be noticed that the criminal could not reach the ABC gate, staying away from it. These artifacts are commonly used in face presentation attacks according to the bibliography [10,40]:

- Printed photo attacks with a high resolution
- Printed photomask
- Printed eyeless photomask (simulating a real human being performing eye blinking)
- A high-resolution image displayed on a tablet

The present research work is focused on results obtained in close to real situations. Therefore, experiments were developed in two locations (Adolfo Suarez-Barajas Airport in Madrid and Algeciras Port in Cadiz) in real border crossings and operational environments. Close interaction with the border guard was mandatory to carry out this study.

**Figure 1:**
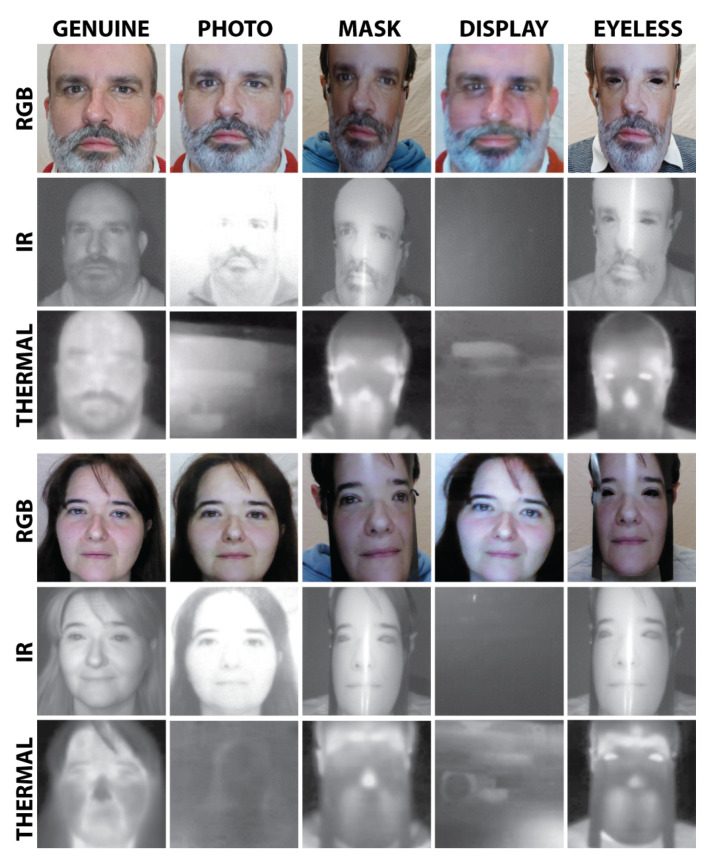
An example of visible lights bona fide or genuine user and its corresponding attacks. The pictures are genuine user, photo attack, paper mask, paper mask with the eye holes cropped, and tablet attack.

All pictures in corpus follow the standard ICAO Doc 9303 (see [43]) in which camera sensor distance (CSD) is recommended as 1 m <=CSD<=2.5 m. As shown in Figure 1, all different kinds of lights such as visible, near-infrared, and thermal are depicted.

The face has been detected and cropped in samples because databases are mainly composed of facial images, similar to previous studies [35,37]. The three particular subsets (VIS, NIR, and thermal) were acquired from the same eGate but with different sensors. Images were processed independently and resized to 128 × 128 pixels.

**Figure 2:**
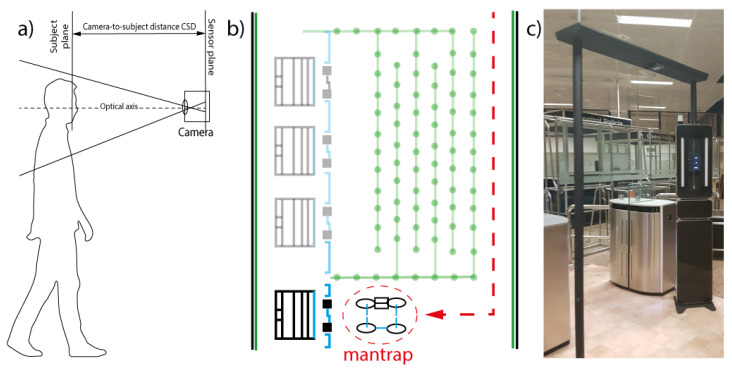
(**a**) The acquisition scheme; (**b**) border point representation with the dedicated gate; and (**c**) a real ABC system.

According to the scheme in Figure 2, the corpus was acquired. Firstly, Figure 2a depicts a subject approaching sensors. Following, Figure 2b describes the deployment of the system at the border. The passenger crosses the system, images are taken and processed, detecting the criminals in situ. Finally, Figure 2c illustrates the system showing the passengers’ approach at the border.

## 4. Method and Experimental Description

### 4.1. Method

The general structure of the developed experiments is a convolutional neural network followed by different classifiers. Once the CNN is done, the most suitable image features are learned, and then, a feature vector is returned. As an input to the CNN, three subsets of the database (described in the previous section) are used following different information fusion approaches: (1) visible (VIS), NIR, and thermal separately; (2) VIS, NIR, and thermal mixed and added at the characteristic level; and (3) the three subsets mixed and added at the classification level.

**Figure 3:**
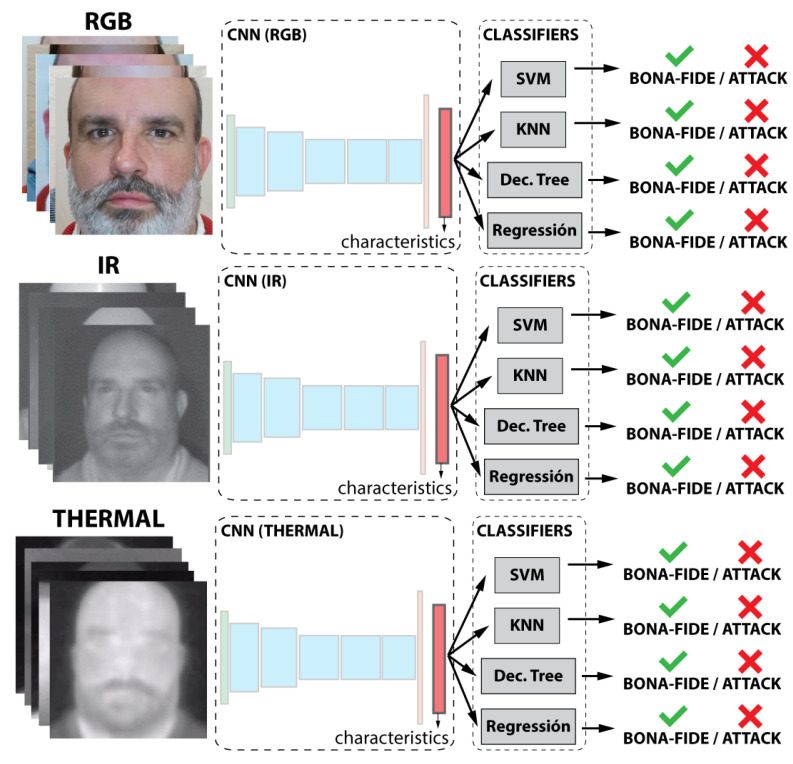
Representation of the first case of study.

As shown in Figure 3, the classification task is performed by five different classifiers. The selected classifiers are support vector machine (SVM) with radial basis function (RBF) and linear kernel, k-nearest neighbor (KNN), decision tree, and logistic regression. Each classifier was trained independently.

#### 4.1.1. CNN Architecture

The architecture of the convolutional neural network is based on AlexNet [36] with some adaptations to the current problem to improve the performance. The layers considered are the following:

1. Convolutional Layer (11 × 11) + MaxPool layer (2 × 2) + Normalization layer
2. Convolutional Layer (4 × 4)
3. Convolutional Layer (3 × 3)
4. Convolutional Layer (3 × 3)
5. Convolutional Layer (3 × 3) + MaxPool layer (2 × 2)
6. Dropout Layer
7. Dropout Layer
8. Fully connected Layer

**Figure 4:**
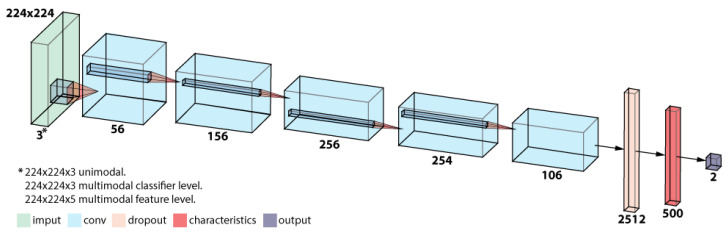
Network architecture.

The number of kernels used for each convolutional layer is 56 for the first layer, 156 for the second, 256 for the third, 254 for the fourth, and 106 for the last convolutional layer. At the dropout layer, 2512 neurons were used, and 500 were fully connected (see Figure 4).

A rectified linear unit (ReLU) was used as the activation function in every convolutional and fully connected layer. The logistic regression function was used to train the network; the negative likelihood was used to compute the cost function during training. Regarding the initialization weights, Gaussian distribution (with std = 0.01 and mean value = 0) and uniform distribution for initialization (also called “Xavier initialization”) were tested. The best performance obtained was with the Gaussian initialization weight.

Different learning rates were tested: 0.01 static learning rate and a dynamic learning rate with an initial value of 0.0095 and a decay proportional to 0.995 per epoch. The best learning rate configuration used in the described experiments is the dynamic learning rate. Thirty samples were used per batch. The training procedure was run for 200 epochs. The weights of the convolutional layers and the fully connected layers were learned and adapted to this approach. The code was developed using the Theano framework [44] to optimize the model on the GPU.

#### 4.1.2. Classification

Samples were tagged as 0 or 1, depending on whether samples belong to bona fide or criminal cases. Thus, it is a dichotomic classification task, and no distinction is realized among attacks. As a result, the number of negative samples was higher than the number of positive samples. After the CNN training procedure, classifiers were trained and validated, obtaining the hyperparameters that are better suited in the classification task.

### 4.2. Experimental Description

To test the proposed system, a complete experiment set was designed. The different scenarios are presented as follows. The first is individual evaluation, and then, two approaches are explained: concatenating the three databases before the CNN procedure (at the feature level) and concatenating the feature vectors after each independent CNN procedure (at the classification level).

The corpus was divided into two parts. The first part was composed of 185 bona fide pictures/subjects. On the other hand, a combination of the number of attacks (four) and bona fide pictures returned 740 attacks’ pictures. In its turn, CNN’s training dataset was split into two subsets (training and validation). Eighty-five percent of pictures were used for the training process and the remaining pictures 15% were selected for the validation process. Thus, 157 out of 185 of bona fide pictures and 629 out of 740 attack pictures were used to training the subset. Likewise, 28 out of 185 bona fide pictures and 111 out of 740 were selected for the validation process.

Regarding classifiers’ training, it should be noticed that all pictures were used for this process, and image distribution was unbalanced. To avoid this fact, it was mandatory to carry out a resample method. This method was based on the use of the k-fold cross-validation process, as described in [45]. The two subsets, training and test, were built n times. Random training and test samples were selected out of total samples, following the proportion 75% for the training and 25% for the test. Then, the Attack Presentation Classification Error Rate (APCER) and Bona fide Presentation Classification Error Rate (BPCER) were calculated. As might be expected, *K* equal to 5 is adequate to avoid this unbalance.

The evaluation of a PAD system has been described in recent years using different measurements, but the community has achieved a common point of view with the definition of the standard ISO (IEC 30107-3:2016). In this standard, the attack capability detection is measured with errors: attack presentation classification error rate (APCER), bona fide presentation classification error rate (BPCER), and average classification error rate (ACER), as defined below.

- **Attack Presentation Classification Error Rate (APCER)** is defined as the proportion of presentation attacks that were classified incorrectly (as bona fide) [46] (Equation (1)).
  (1) $$APCER_{PAIs}=1-\left(\frac{1}{\left|PAI\right|}\right)\sum_{\omega =1}^{\left|PAI\right|}\left(RES_{\omega }\right),$$
  where |PAI| is the number of presentation attack instruments (PAI) and $RES_{\omega }$ takes the value 1 if the presentation ω is assessed as an attack and 0 if it is evaluated as bona fide. A PAI is defined as an object or biometric trait used in a presentation attack.
- **Bona fide Presentation Classification Error Rate (BPCER)** is defined as the proportion of bona fide presentation incorrectly classified as presentation attacks [46] (Equation (2)).
  (2) $$BPCER_{PAIs}=\frac{\sum_{\omega =1}^{\left|BF\right|}RES_{\omega }}{\left|BF\right|},$$
  where |BF| is the cardinal of bona fide presentations and $RES_{i}$ returns the value 1 if the presentation ω is allocated as ab attack and 0 if it is analyzed as bona fide.
- **Average Classification Error Rate (ACER)**: is weighted average between APCER and BPCER
  (3) $$ACER_{PAIs}=\frac{APCER_{PAIs}+BPCER_{PAIs}}{2},$$

#### 4.2.1. First Case of Study—Unimodal Evaluation

In this experiment, the objective was to evaluate the performance using each kind of information individually, so each subset (visible, thermal, and NIR) was **tested separately**. Each subset is used to independently train a CNN and the classifiers. In this case, three different and independent results were obtained, one per subset (see Figure 3).

#### 4.2.2. Second Case of Study—Classifier-Level Multimodal Fusion

**Figure 5:**
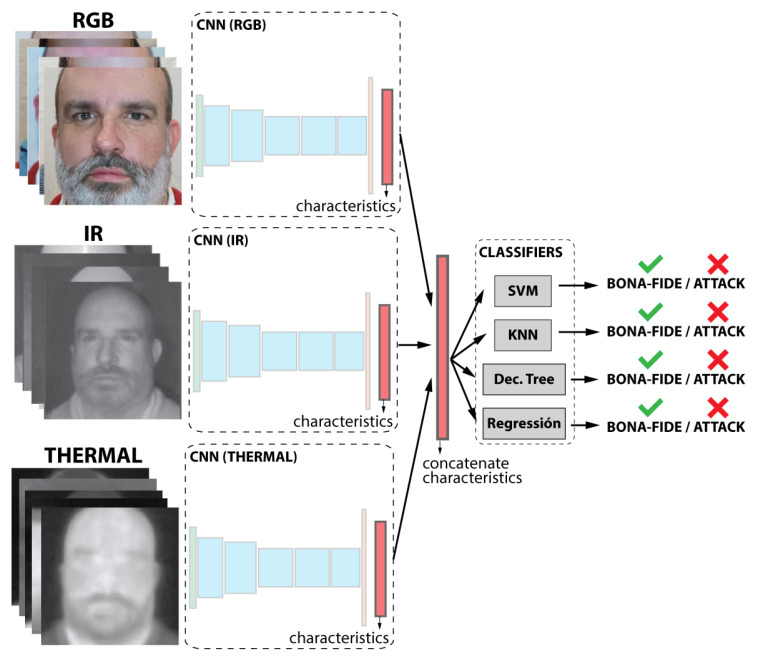
Representation of the second case of study. Classifier-level multimodal fusion.

In this experiment, each subset was used to train a separate CNN; therefore, three CNNs were trained with the same architecture but different training data. Each neural network was trained with a kind of image (VIS, NIR, or thermal). The outputs of three neural networks were concatenated in a characteristic vector, whose dimension is the sum of the three-dimensional subsets, as represented in Figure 5. A concatenated vector was fed to the classifiers. As in the first case, classifiers were then trained.

#### 4.2.3. Third Case of Study—Feature-Level Multimodal Fusion

**Figure 6:**
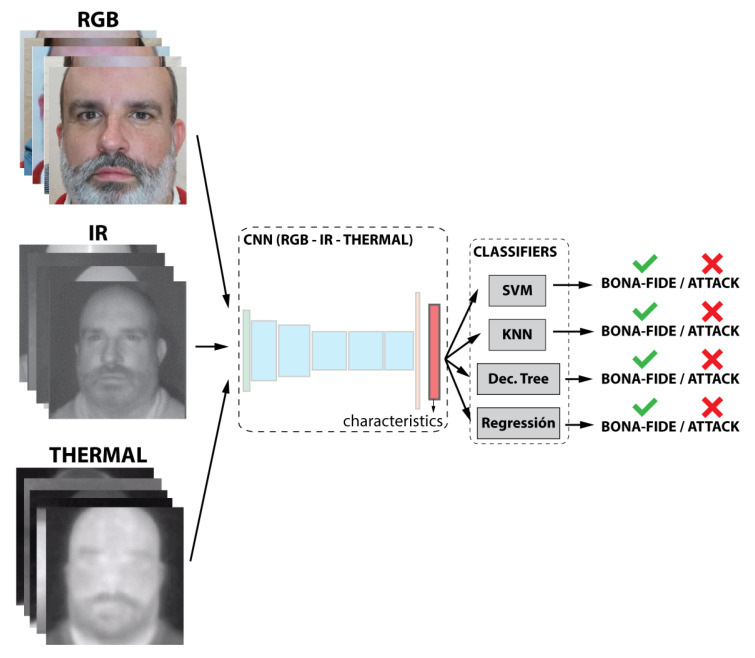
Representation of the third case of study. Feature-level multimodal fusion.

This experiment describes the feature level. Visible light images provide three different channels. The first channel is for the red color (R), the second channel is for the green color (G), and the last channel is for the blue color (B). However, thermal and NIR images present only a gray-scale channel; for this reason, these kinds of images contribute as a unique additional channel to each one. Thus, the resulting output of concatenating images of three different subsystems returns a five-channel image (R, G, B, NIR, and Thermal), as depicted in Figure 6. This image composed of five channels (R, G, B, NIR, and Thermal) is the input to the neural network.

In summary, five results were obtained: visible, NIR, and thermal (independently); the three databases added at the feature level; and the three databases added at the classification level.

## 5. Results and Discussion

### 5.1. Results for Unimodal Evaluation

The model was tested separately for each subset (visible, NIR, and thermal). The results are summarized in Table 1, showing the APCER and BPCER parameters obtained with the best performance of each classifier.

As shown in the output table, some details might be explained in detail, but, overall, the NIR and thermal results obtained were better than the results achieved with the visible sensor. For NIR, all positive samples (BPCER) were correctly classified, whereas the number of negative samples (APCER) incorrectly classified was very low. Considering thermal images, almost 100% percent accuracy was achieved for attacks, and the number of positive samples wrongly classified was very low. The visible sensor had high performance, and the results obtained showed a good balance in comparison to the other sensors. It is worth stressing that the thermal sensor is the best fitted for high-security scenarios since attackers are correctly classified. The tradeoff of thermal sensors is, in some cases, a bona fide passenger being detected as a criminal, and the cost in this situation is associated with disturbing passengers and border guards devoted to processing false positives. In addition, thermal sensors are usually more expensive than visual sensors. However, the NIR sensor is the best fitted for friendly environments in which the low risk is a guarantee since bona fide passengers are always correctly classified. NIR outcomes show that some criminals may be labeled as bona fide passengers. In low-risk situations, a decision can be made from images obtained by this sensor since border guards will not be interrupted with false positives. The visible sensor is a good compromise among both options. The results from both APCER and BPCER are kept low; therefore, the classifier behavior is very uniform in all scenarios. Indeed, the visible sensor has a remarkable advantage in ABC situations because images provided for the sensor can be directly sent to the border guard. Subsequently, the cost of acquisition and replacement of visible sensors are negligible compared with the cost of the other sensors.

The classifier results highly depend on the dataset selected. Regarding visible images, KNN and SVM obtain the same result; thus, both can be considered the best classifiers. Perhaps the main reason is based on CNN returning a good set of features, and a simple classifier such as KNN can obtain a good classification score. Increasing the complexity of the algorithm using a decision tree or logistic regression will not improve the scores obtained previously. Similar comments related to the thermal or NIR images can be made. Finally, in the thermal sensor, the SVM classifier obtains a slightly better result than KNN.

### 5.2. Results for Classifier-Level Fusion

The model was tested when one image from each sensor was analyzed with one specific CNN and the three outputs were concatenated. This new vector is the input of the classifier stage, in which five classifiers are tested. The outcomes achieved from each classifier are summarized in Table 2. In most of the classifiers, attack samples were classified accurately, and the mistaken classified samples correspond to genuine users. Therefore, these results show the potential use in environments in which security is the main objective. In an ABC, when the security level is high, this kind of fusion can be considered. The only exception to these results is the decision tree algorithm that provides the opposite behavior. For this situation, linear SVM outperforms KNN and radial basis function SVM. Note that linear SVM and logistic regression accomplish the same performance. Comparing the scores in Table 2 with the unimodal evaluation in Table 1, it can be seen that adding more information to the classifiers results in better performance. The main disadvantage of this fusion is mainly due to the cost of adding three sensors to the ABC gate. More sensors increase the budget of the overall system and maintain higher maintenance expenses.

### 5.3. Results for Feature-Level Fusion

The last cluster of outcomes corresponds to the three subsets added at the feature level, that is to say, images are concatenated and then fed into a CNN. The performance is depicted in Table 3 for each classifier. Bona fide passengers are always correctly classified, whereas, in some cases, criminals are incorrectly classified as bona fide passengers. This result discourages the use of this fusion method for a situation in which high security is required. This fusion can be used in normal or permissive situations, in which the main goal is to maintain a fluid passenger flow. The best result is obtained with the logistic regression algorithm. KNN and SVM classifiers achieve similar results. Comparing results between NIR unimodal and feature-level fusion, it can be noticed that the outputs are very similar in this particular case. Therefore, the additional study case does not seem to improve the unimodal results.

### 5.4. Discussion

The proposed approach was assessed with different sensors and acquisition systems close-to-real ABC scenarios. The results accomplished with cameras, in the visible range, are a good solution for ABC in normal operating situations. Considering high-security scenarios, the thermal sensor shows the best behavior since all criminals are correctly classified. The fusion of the different images shows that classifier-level fusion is the best fit for high-security scenarios, whereas, in a relaxed security operation, feature-level fusion seems to be an adequate option. The use of all information sources provides better performance in most cases, using the classifier or feature-level fusion. However, using NIR information individually, feature-level fusion does not seem to improve the unimodal performance. Finally, classifiers such as KNN or SVM show discriminative power, keeping enough low complexity.

Comparing the outcomes attained with similar results in the literature, it seems that these are competitive. In Table 4, an exhaustive comparison between the current approach and a selection of recent research works with similar experiments is shown. It is not a straightforward issue selection of studies with similar datasets and evaluation conditions. Some key points to take into account are evaluation metrics, corpora, number of attacks, and sensors involved. Moreover, not all research works have adopted standard ISO metrics [12], which has been established, recently. Likewise, in most cases, the used corpora have been acquired in a controlled environment, whereas the present study obtained all pictures in a real scenario. Finally, two recent research works could be considered as studies with similar conditions [26,47].

Contrasting RGB or gray-scale images performance, it can be observed which the presented research work decreases, one order of magnitude, APCER value (from 40.3 in [47], and 65.65 in [26] to 2.45 APCER in the current approach. In contrast, in the case of BPCER, the present study increases BPCER results. This consequence is logical due to a high-security environment being an ABC border. This environment is mainly designed to avoid APCER. To sum up, the mean ACER is reduced to 11.8, a better result than in two other studies.

Regarding IR outcomes, the current approach improves on the selected previous studies. The APCER is decreased from 5.03 [26] to 1.23, whereas the attained BPCER achieves 0 in both cases. Focusing on thermal results, only George [26] presented this kind of sensor. Once again, the current study shows a remarkable reduction of APCER (0 versus 3.15) but increasing BPCER (4.26 versus 0.56) and ACER.

Finally, analyzing the presented fusion results with the selected works, the authors have chosen two different configurations in their current approach. On the one hand, Log. Regression (Classifier-Level Fusion) attained the lowest APCER and Log. Regression (Feature-Level Fusion) accomplished the lowest BPCER. On the other hand, the first approach returns very similar values to the study by George [26], in which APCER is 0.6, and ACER is 0.3 in both cases. In the second study, the outcomes obtain 0 APCER, increasing BPCER from 0 to 2.13.

## 6. Conclusions and Future Work

This paper proposes a close-to-real use of facial presentation attack detection in border control. CNNs were considered to learn features for multispectral face presentation attack detection (visible, near-infrared, and thermal). Five different classifiers were used (linear and RBF SVM, KNN, decision tree, and logistic regression).

Four different attacks were considered using the following artifacts: printed photo, printed mask, printed mask with the eyes cropped, and an image displayed on a tablet. Considering the experimental results, it seems that the CNN paradigm is optimal for presentation attack detection, obtaining relevant results.

In normal operational situations for ABC systems, visible images can be considered a good option, as shown in the results, and considering that they are usually the main or only acquisition sensors. For high-security situations, the thermal sensor shows better performance since all attackers are correctly classified, whereas in only a few cases can a bona fide passenger be detected as a criminal. The opposite situation is achieved in the case of the NIR sensor: all bona fide travelers are correctly categorized, but some attacks are undetected and wrongly classified as bona fide.

The fusion of the different images shows that classifier-level fusion is the best fit for high-security scenarios. However, in a permissive security operation, feature-level fusion seems to be the best fit. Using all information sources shows better performance than the use of isolated sensors, in both the classifier are the feature-level fusions. Finally, classifiers such as KNN or SVM present enough discriminative power to maintain low complexity.

## Figures and Tables

**Table 1:** Unimodal results.

	Visible			NIR			Thermal		
	**APCER** **(%)**	**BPCER** **(%)**	**ACER** **(%)**	**APCER** **(%)**	**BPCER** **(%)**	**ACER** **(%)**	**APCER** **(%)**	**BPCER** **(%)**	**ACER** **(%)**
SVM RBF	2.45	23.40	12.9	1.23	0	0.61	0	6.38	3.19
SVM Linear	2.45	21.27	11.8	1.23	0	0.61	0	6.38	3.19
KNN	2.45	21.27	11.8	1.23	0	0.61	1.84	6.38	4.11
Dec. Tree	4.29	21.27	12.7	18.4	0	9.2	0	8.51	4.25
Log. Regression	3.06	7.41	5.23	18.4	0	9.2	0	4.26	2.13

**Table 2:** Classifier-level fusion.

	APCER (%)	BPCER (%)	ACER (%)
SVM RBF	0	6.38	3.19
SVM Linear	0	2.13	1.06
KNN	0	4.26	2.13
Dec. Tree	1.84	0	0.92
Log. Regression	0	2.13	1.06

**Table 3:** Feature-level fusion.

	APCER (%)	BPCER (%)	ACER (%)
SVM RBF	1.23	0	0.61
SVM Linear	1.23	0	0.61
KNN	1.23	0	0.61
Dec. Tree	1.84	0	0.92
Log. Regression	0.61	0	0.31

**Table 4:** Comparison between current study outcomes and other research works.

Database	#Subjects/#Attacks	Algorithm	Sensor	APCER(%)	BPCER(%)	ACER(%)
**CASIA-SURF [47]**	1000/Pictures and masks	based RESNET-18	RGB	40.3	1.6	21.0
			Depth	6.0	1.2	3.6
			IR	38.6	0.4	19.4
			RGB + Depth	5.8	0.8	3.3
			RGB + IR	36.5	0.005	18.3
			Depth + IR	2.0	0.3	1.1
			RGB + Depth + IR	1.9	0.1	1.0
**WMCA** [26]	72/Pictures, glasses, replay, and masks	MC-CNN (Multi-Channel Convolutional Neural Network)	Grayscale + Depth + IR + Thermal	0.6	0	0.3
			Grayscale + Depth + IR	2.07	0	1.04
			Grayscale	65.65	0	32.82
			Depth	11.77	0.31	6.04
			IR	5.03	0	2.51
			Thermal	3.14	0.56	1.85
**FRAV-ATTACK**	Current study	SVM RBF	RGB	2.45	23.4	12.9
		**SVM RBF**	**IR**	**1.23**	**0**	**0.61**
		SVM RBF	Thermal	0	6.38	3.19
		SVM RBF (Classifier-Level Fusion)	RGB + IR + Thermal	0	6.38	3.19
		SVM RBF (Feature-Level Fusion)	RGB + IR + Thermal	1.23	0	0.61
		**SVM Linear**	**RGB**	**2.45**	**21.27**	**11.8**
		**SVM Linear**	**IR**	**1.23**	**0**	**0.61**
		SVM Linear	Thermal	0	6.38	3.19
		SVM Linear (Classifier-Level Fusion)	RGB + IR + Thermal	0	2.13	1.06
		SVM Linear (Feature-Level Fusion)	RGB + IR + Thermal	1.23	0	0.61
		**KNN**	**RGB**	**2.45**	**21.27**	**11.8**
		**KNN**	**IR**	**1.23**	**0**	**0.61**
		KNN	Thermal	1.84	6.38	4.11
		KNN (Classifier-Level Fusion)	RGB + IR + Thermal	0	4.26	2.13
		KNN (Feature-Level Fusion)	RGB + IR + Thermal	1.23	0	0.61
		Dec. Tree	RGB	4.29	21.27	12.7
		Dec. Tree	IR	18.4	0	9.2
		Dec. Tree	Thermal	0	8.51	4.25
		Dec. Tree (Classifier-Level Fusion)	RGB + IR + Thermal	1.84	0	0.92
		Dec. Tree (Feature-Level Fusion)	RGB + IR + Thermal	1.84	0	0.92
		Log. Regression	RGB	3.06	7.41	5.23
		Log. Regression	IR	18.4	0	9.2
		**Log. Regression**	**Thermal**	**0**	**4.26**	**2.13**
		**Log. Regression** **(Classifier-Level Fusion)**	**RGB + IR + Thermal**	**0**	**2.13**	**1.06**
		**Log. Regression** **(Feature-Level Fusion)**	**RGB + IR + Thermal**	**0.61**	**0**	**0.31**
